# *N*′-[(*E*)-2-Hy­droxy-3,5-diiodo­benzyl­idene]cyclo­hexa­ne-1-carbohydrazide

**DOI:** 10.1107/S1600536811036488

**Published:** 2011-09-14

**Authors:** A. Thirugnanasundar, J. Suresh, C. Meenakshi, G. Chakkaravarthi, G. Rajagopal

**Affiliations:** a Department of Chemistry, Velalar College of Engineering and Technology, Erode 638 009, India; b Department of Physics, The Madura College, Madurai 625 011, India; c Department of Chemistry, Government Arts College for Women (Autonomous), Madurai 625 002, India; d Department of Physics, CPCL Polytechnic College, Chennai 600 068, India; e Department of Chemistry, Government Arts College, Melur 625 106, India

## Abstract

In the title compound, C_14_H_10_I_2_N_2_O_2_, the two aromatic rings are inclined at a dihedral angle of 16.72 (33)°. The mol­ecular structure is stabilized by an intra­molecular O—H⋯N hydrogen bond. In the crystal, inter­molecular N—H⋯O inter­actions link the mol­ecules into chains running along the *c* axis. C—H⋯O inter­actions also occur. The crystal used for the structure determination was a non-merohedral twin with a domain ratio of 0.972 (2):0.028 (2).

## Related literature

For the biological activity of Schiff base derivatives, see: Daier *et al.* (2004 ▶); Golcu *et al.* (2005 ▶); Liu & Yang (2010 ▶); Zgierski & Grabowska (2000 ▶). For related structures, see: Manvizhi *et al.* (2011 ▶); Thirugnanasundar *et al.* (2011 ▶).
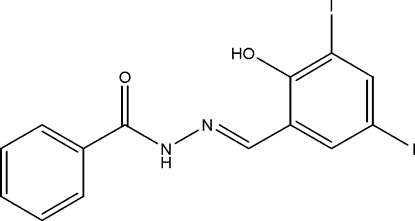


## Experimental

### 

#### Crystal data

C_14_H_10_I_2_N_2_O_2_
*M* *_r_* = 492.04
Monoclinic, 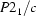

*a* = 17.7495 (13) Å
*b* = 9.4273 (6) Å
*c* = 9.4684 (7) Å
β = 103.052 (3)°
*V* = 1543.42 (19) Å^3^
*Z* = 4
Mo *K*α radiation
μ = 4.08 mm^−1^
*T* = 295 K
0.20 × 0.10 × 0.10 mm

#### Data collection

Bruker Kappa APEXII diffractometer
Absorption correction: multi-scan (*SADABS*; Sheldrick, 1996 ▶) *T*_min_ = 0.496, *T*_max_ = 0.686
13146 measured reflections
2708 independent reflections
2349 reflections with *I* > 2σ(*I*)
*R*_int_ = 0.029

#### Refinement

*R*[*F* ^2^ > 2σ(*F* ^2^)] = 0.058
*wR*(*F* ^2^) = 0.172
*S* = 1.11
2708 reflections
182 parameters
H-atom parameters constrained
Δρ_max_ = 1.48 e Å^−3^
Δρ_min_ = −1.52 e Å^−3^

### 

Data collection: *APEX2* (Bruker, 2004 ▶); cell refinement: *SAINT* (Bruker, 2004 ▶); data reduction: *SAINT*; program(s) used to solve structure: *SHELXS97* (Sheldrick, 2008 ▶); program(s) used to refine structure: *SHELXL97* (Sheldrick, 2008 ▶); molecular graphics: *PLATON* (Spek, 2009 ▶); software used to prepare material for publication: *SHELXL97*.

## Supplementary Material

Crystal structure: contains datablock(s) global, I. DOI: 10.1107/S1600536811036488/bt5637sup1.cif

Structure factors: contains datablock(s) I. DOI: 10.1107/S1600536811036488/bt5637Isup2.hkl

Supplementary material file. DOI: 10.1107/S1600536811036488/bt5637Isup3.cml

Additional supplementary materials:  crystallographic information; 3D view; checkCIF report

## Figures and Tables

**Table 1:** Hydrogen-bond geometry (Å, °)

*D*—H⋯*A*	*D*—H	H⋯*A*	*D*⋯*A*	*D*—H⋯*A*
O2—H2*A*⋯N2	0.85	1.84	2.586 (13)	145
N1—H1⋯O1^i^	0.86	2.02	2.824 (14)	155
C3—H3⋯O2^ii^	0.93	2.53	3.365 (19)	150

Symmetry codes: (i) 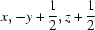; (ii) 
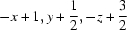
.

**Fig. 1.**
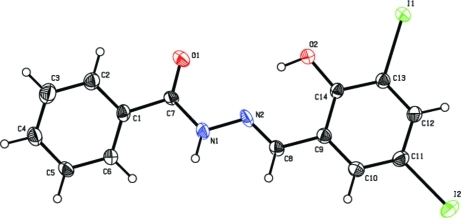
The molecular structure of (I), with atom labels and 30% probability displacement ellipsoids for non-H atoms.

**Fig. 2.**
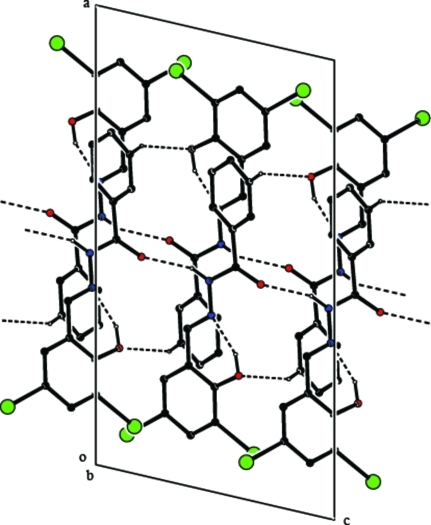
The packing of (I), viewed down b axis. Intermolecular Hydrogen bonds are shown as dashed lines. H atoms not involved in hydrogen bonding have been omitted.

## supplementary crystallographic information

### Comment

Schiff base ligands play an important role in the development of coordination chemistry and possess important properties such as biological activity (Golcu  *et al.*, 2005; Liu & Yang, 2010), catalytic activity (Daier *et al.*, 2004) and photochromic properties (Zgierski & Grabowska, 2000).

The goemetric parameters of the title compound   (Fig. 1)   agree well with   related structures (Manvizhi *et al.*, 2011; Thirugnanasundar *et al.*, 2011).   The two aromatic rings are inclined at an angle of 16.72 (33)°.

The molecular structure is stabilized by an intramolecular O-H···N hydrogen bond. The crystal structure is controlled by intermolecular N-H···O and C—H···O (Fig. 2 & Table 1) interactions.

### Experimental

A methanolic solution (10 ml) of  benzoicacid hydrazide (5 mmol) was magnetically stirred in a round bottom flask followed by drop wise addition of  methanolic solution of  3,5-diiodosalicylaldehyde (5 mmol). The reaction mixture was then refluxed for three hours and upon cooling to 278 K, a  yellow crystalline solid precipitates from the mixture. The solid which is separated out was filtered washed with ice cold ethanol and dried in vaccuo over anhydrous CaCl_2_. Single crystals suitable for the X-ray diffraction were obtained by slow evaporation of a solution of the title compound in DMF at room temperature. Melting Point : 485 K.

### Refinement

All H atoms were positioned geometrically with C—H = 0.93Å, O—H = 0.82 Å and N—H = 0.86 Å and allowed to ride on their parent atoms, with Uiso(H) = 1.5 Ueq(O), 1.2 Ueq(N) and 1.2 Ueq(C). Initial checkCIF/PLATON results indicated possible twinning; introduction of the suggested command  TWIN 1 0 0.85 0 -1 0 0 0 -1  2 during refinement gave a very modest decrease in the R-factor, from 0.0743 to 0.0584, with BASF = 0.028 (2).

### Crystal data

**Table:**

C_14_H_10_I_2_N_2_O_2_	*F*(000) = 920
*M**_r_* = 492.04	*D*_x_ = 2.118 Mg m^−^^3^
Monoclinic, *P*2_1_/*c*	Mo *K*α radiation, λ = 0.71073 Å
Hall symbol: -P 2ybc	Cell parameters from 13146 reflections
*a* = 17.7495 (13) Å	θ = 2.2–25.0°
*b* = 9.4273 (6) Å	µ = 4.08 mm^−^^1^
*c* = 9.4684 (7) Å	*T* = 295 K
β = 103.052 (3)°	Block, yellow
*V* = 1543.42 (19) Å^3^	0.20 × 0.10 × 0.10 mm
*Z* = 4	

### Data collection

**Table:**

Bruker Kappa APEXII diffractometer	2708 independent reflections
Radiation source: fine-focus sealed tube	2349 reflections with *I* > 2σ(*I*)
graphite	*R*_int_ = 0.029
ω and φ scans	θ_max_ = 25.0°, θ_min_ = 2.2°
Absorption correction: multi-scan (*SADABS*; Sheldrick, 1996)	*h* = −21→21
*T*_min_ = 0.496, *T*_max_ = 0.686	*k* = −11→8
13146 measured reflections	*l* = −11→11

### Refinement

**Table:**

Refinement on *F*^2^	Primary atom site location: structure-invariant direct methods
Least-squares matrix: full	Secondary atom site location: difference Fourier map
*R*[*F*^2^ > 2σ(*F*^2^)] = 0.058	Hydrogen site location: inferred from neighbouring sites
*wR*(*F*^2^) = 0.172	H-atom parameters constrained
*S* = 1.11	*w* = 1/[σ^2^(*F*_o_^2^) + (0.0412*P*)^2^ + 42.3729*P*] where *P* = (*F*_o_^2^ + 2*F*_c_^2^)/3
2708 reflections	(Δ/σ)_max_ < 0.001
182 parameters	Δρ_max_ = 1.48 e Å^−^^3^
0 restraints	Δρ_min_ = −1.52 e Å^−^^3^

### Fractional atomic coordinates and isotropic or equivalent isotropic displacement parameters (Å^2^)

**Table:**

	*x*	*y*	*z*	*U*_iso_*/*U*_eq_	
I1	0.89740 (5)	0.16968 (10)	0.83785 (10)	0.0458 (3)	
I2	0.91863 (7)	−0.18253 (14)	1.36905 (13)	0.0777 (5)	
O1	0.5247 (5)	0.3352 (11)	0.8089 (10)	0.052 (3)	
O2	0.7346 (5)	0.2255 (10)	0.9018 (10)	0.044 (2)	
H2A	0.6895	0.2404	0.9145	0.065*	
N1	0.5428 (6)	0.2182 (13)	1.0250 (11)	0.043 (3)	
H1	0.5244	0.1906	1.0969	0.052*	
N2	0.6169 (6)	0.1863 (12)	1.0158 (11)	0.040 (3)	
C1	0.4189 (7)	0.3296 (14)	0.9272 (12)	0.036 (3)	
C2	0.3821 (7)	0.4436 (15)	0.8509 (13)	0.043 (3)	
H2	0.4075	0.4988	0.7950	0.052*	
C3	0.3079 (9)	0.4755 (18)	0.8577 (16)	0.057 (4)	
H3	0.2836	0.5541	0.8082	0.068*	
C4	0.2694 (8)	0.3939 (16)	0.9356 (15)	0.047 (3)	
H4	0.2185	0.4156	0.9369	0.057*	
C5	0.3052 (7)	0.2777 (15)	1.0138 (13)	0.041 (3)	
H5	0.2790	0.2217	1.0678	0.049*	
C6	0.3807 (7)	0.2479 (15)	1.0092 (14)	0.041 (3)	
H6	0.4060	0.1719	1.0621	0.049*	
C7	0.4996 (7)	0.2964 (14)	0.9137 (11)	0.035 (3)	
C8	0.6579 (7)	0.1120 (14)	1.1159 (12)	0.036 (3)	
H8	0.6375	0.0800	1.1924	0.043*	
C9	0.7375 (7)	0.0770 (13)	1.1107 (13)	0.034 (3)	
C10	0.7797 (7)	−0.0131 (15)	1.2142 (14)	0.042 (3)	
H10	0.7576	−0.0486	1.2872	0.051*	
C11	0.8534 (8)	−0.0503 (15)	1.2102 (14)	0.043 (3)	
C12	0.8881 (7)	0.0029 (13)	1.1027 (14)	0.040 (3)	
H12	0.9385	−0.0225	1.1010	0.048*	
C13	0.8471 (7)	0.0932 (13)	0.9997 (13)	0.034 (3)	
C14	0.7710 (6)	0.1333 (13)	1.0022 (12)	0.031 (2)	

### Atomic displacement parameters (Å^2^)

**Table:**

	*U*^11^	*U*^22^	*U*^33^	*U*^12^	*U*^13^	*U*^23^
I1	0.0346 (4)	0.0620 (6)	0.0442 (5)	0.0048 (4)	0.0159 (4)	0.0154 (4)
I2	0.0733 (8)	0.0933 (9)	0.0714 (8)	0.0406 (7)	0.0266 (6)	0.0484 (7)
O1	0.041 (5)	0.074 (7)	0.047 (6)	0.002 (5)	0.021 (4)	0.010 (5)
O2	0.034 (4)	0.048 (5)	0.051 (5)	0.007 (4)	0.015 (4)	0.015 (4)
N1	0.032 (5)	0.064 (7)	0.036 (6)	0.015 (5)	0.015 (4)	0.005 (5)
N2	0.024 (5)	0.058 (7)	0.042 (6)	0.002 (5)	0.017 (4)	−0.006 (5)
C1	0.026 (6)	0.053 (8)	0.025 (6)	0.003 (5)	−0.001 (5)	−0.006 (5)
C2	0.041 (7)	0.054 (8)	0.034 (7)	0.006 (6)	0.006 (6)	0.000 (6)
C3	0.052 (9)	0.066 (10)	0.051 (8)	0.018 (8)	0.010 (7)	0.002 (8)
C4	0.036 (7)	0.055 (9)	0.054 (8)	0.007 (6)	0.016 (6)	−0.002 (7)
C5	0.035 (6)	0.059 (8)	0.033 (6)	0.003 (6)	0.016 (5)	0.003 (6)
C6	0.034 (6)	0.046 (8)	0.046 (7)	0.008 (6)	0.013 (6)	0.010 (6)
C7	0.031 (6)	0.057 (8)	0.017 (5)	0.001 (5)	0.007 (4)	0.001 (5)
C8	0.034 (6)	0.052 (8)	0.023 (6)	0.005 (6)	0.011 (5)	−0.005 (5)
C9	0.034 (6)	0.039 (7)	0.031 (6)	0.003 (5)	0.010 (5)	−0.002 (5)
C10	0.042 (7)	0.053 (8)	0.036 (7)	0.007 (6)	0.016 (6)	0.006 (6)
C11	0.046 (7)	0.048 (8)	0.034 (6)	0.007 (6)	0.008 (6)	0.006 (6)
C12	0.042 (7)	0.037 (7)	0.044 (7)	0.011 (6)	0.016 (6)	0.004 (5)
C13	0.033 (6)	0.037 (7)	0.032 (6)	0.003 (5)	0.008 (5)	−0.002 (5)
C14	0.031 (6)	0.033 (6)	0.028 (6)	0.006 (5)	0.006 (5)	−0.003 (5)

### Geometric parameters (Å, °)

**Table:**

I1—C13	2.070 (12)	C4—C5	1.393 (19)
I2—C11	2.090 (13)	C4—H4	0.9300
O1—C7	1.231 (14)	C5—C6	1.379 (17)
O2—C14	1.342 (14)	C5—H5	0.9300
O2—H2A	0.8480	C6—H6	0.9300
N1—C7	1.370 (15)	C8—C9	1.463 (16)
N1—N2	1.370 (13)	C8—H8	0.9300
N1—H1	0.8600	C9—C10	1.382 (17)
N2—C8	1.268 (16)	C9—C14	1.402 (16)
C1—C2	1.375 (18)	C10—C11	1.363 (18)
C1—C6	1.376 (18)	C10—H10	0.9300
C1—C7	1.500 (16)	C11—C12	1.397 (18)
C2—C3	1.366 (19)	C12—C13	1.373 (17)
C2—H2	0.9300	C12—H12	0.9300
C3—C4	1.35 (2)	C13—C14	1.409 (16)
C3—H3	0.9300		
			
C14—O2—H2A	108.9	O1—C7—C1	122.2 (11)
C7—N1—N2	116.6 (10)	N1—C7—C1	115.2 (10)
C7—N1—H1	121.7	N2—C8—C9	119.7 (11)
N2—N1—H1	121.7	N2—C8—H8	120.1
C8—N2—N1	118.1 (10)	C9—C8—H8	120.1
C2—C1—C6	119.8 (11)	C10—C9—C14	120.1 (11)
C2—C1—C7	117.9 (12)	C10—C9—C8	119.2 (11)
C6—C1—C7	122.2 (11)	C14—C9—C8	120.8 (11)
C3—C2—C1	119.8 (14)	C11—C10—C9	120.5 (12)
C3—C2—H2	120.1	C11—C10—H10	119.7
C1—C2—H2	120.1	C9—C10—H10	119.7
C4—C3—C2	120.7 (14)	C10—C11—C12	120.8 (12)
C4—C3—H3	119.6	C10—C11—I2	120.9 (10)
C2—C3—H3	119.6	C12—C11—I2	118.3 (9)
C3—C4—C5	120.7 (13)	C13—C12—C11	119.4 (11)
C3—C4—H4	119.6	C13—C12—H12	120.3
C5—C4—H4	119.6	C11—C12—H12	120.3
C6—C5—C4	118.3 (12)	C12—C13—C14	120.7 (11)
C6—C5—H5	120.9	C12—C13—I1	119.6 (9)
C4—C5—H5	120.9	C14—C13—I1	119.7 (9)
C1—C6—C5	120.7 (12)	O2—C14—C9	123.3 (10)
C1—C6—H6	119.7	O2—C14—C13	118.1 (10)
C5—C6—H6	119.7	C9—C14—C13	118.5 (11)
O1—C7—N1	122.6 (11)		
			
C7—N1—N2—C8	−178.8 (12)	N2—C8—C9—C14	4.9 (19)
C6—C1—C2—C3	0(2)	C14—C9—C10—C11	−1(2)
C7—C1—C2—C3	178.5 (12)	C8—C9—C10—C11	178.5 (13)
C1—C2—C3—C4	−2(2)	C9—C10—C11—C12	1(2)
C2—C3—C4—C5	2(2)	C9—C10—C11—I2	178.5 (10)
C3—C4—C5—C6	0(2)	C10—C11—C12—C13	0(2)
C2—C1—C6—C5	1(2)	I2—C11—C12—C13	−178.1 (10)
C7—C1—C6—C5	−177.0 (12)	C11—C12—C13—C14	0.5 (19)
C4—C5—C6—C1	−1(2)	C11—C12—C13—I1	−178.9 (10)
N2—N1—C7—O1	1.0 (19)	C10—C9—C14—O2	−177.3 (12)
N2—N1—C7—C1	−179.9 (11)	C8—C9—C14—O2	2.8 (18)
C2—C1—C7—O1	−22.7 (18)	C10—C9—C14—C13	1.5 (18)
C6—C1—C7—O1	155.4 (13)	C8—C9—C14—C13	−178.4 (11)
C2—C1—C7—N1	158.2 (12)	C12—C13—C14—O2	177.8 (11)
C6—C1—C7—N1	−23.7 (18)	I1—C13—C14—O2	−2.8 (15)
N1—N2—C8—C9	−179.4 (11)	C12—C13—C14—C9	−1.1 (18)
N2—C8—C9—C10	−175.0 (13)	I1—C13—C14—C9	178.3 (9)

### Hydrogen-bond geometry (Å, °)

**Table:**

*D*—H···*A*	*D*—H	H···*A*	*D*···*A*	*D*—H···*A*
O2—H2A···N2	0.85	1.84	2.586 (13)	145
N1—H1···O1^i^	0.86	2.02	2.824 (14)	155
C3—H3···O2^ii^	0.93	2.53	3.365 (19)	150

Symmetry codes: (i) *x*, −*y*+1/2, *z*+1/2; (ii) −*x*+1, *y*+1/2, −*z*+3/2.
